# GFP-LC3 labels organised smooth endoplasmic reticulum membranes independently of autophagy

**DOI:** 10.1002/jcb.22103

**Published:** 2009-03-03

**Authors:** Vladimir M Korkhov

**Affiliations:** MRC Laboratory of Molecular BiologyHills Road, Cambridge CB2 0QH, UK

**Keywords:** GFP-LC3, calnexin, protein aggregate, autophagy, organised smooth ER, multilamellar body

## Abstract

Disruption of autophagy leads to accumulation of intracellular multilamellar inclusions morphologically similar to organised smooth endoplasmic reticulum (OSER) membranes. However, the relation of these membranous compartments to autophagy is unknown. The purpose of this study was to test whether OSER plays a role in the autophagic protein degradation pathway. Here, GFP-LC3 is shown to localise to the OSER membranes induced by calnexin expression both in transiently transfected HEK293 cells and in mouse embryo fibroblasts. In contrast to GFP-LC3, endogenous LC3 is excluded from these membranes under normal conditions as well as after cell starvation. Furthermore, YFP-Atg5, a protein essential for autophagy and known to reside on autophagic membranes, is excluded from the calnexin-positive inclusion structures. In cells devoid of Atg5, a protein essential for autophagy and known to reside on autophagic membranes, colocalisation of calnexin with GFP-LC3 within the multilamellar bodies is preserved. I show that calnexin, a protein enriched in the OSER, is not subject to autophagic or lysosomal degradation. Finally, GFP-LC3 targeting to these membranes is independent of its processing and insensitive to drugs modulating autophagic and lysosomal protein degradation. These observations are inconsistent with a role of autophagic/lysosomal degradation in clearance of multilamellar bodies comprising OSER. Furthermore, GFP-LC3, a fusion protein widely used as a marker for autophagic vesicles and pre-autophagic compartments, may be trapped in this compartment and this artefact must be taken into account if the construct is used to visualise autophagic membranes. J. Cell. Biochem. 107: 86–95, 2009. © 2009 Wiley-Liss, Inc.

Fluorescent protein fusions have greatly facilitated modern science, providing invaluable tools for studying the biogenesis, folding, interactions, trafficking, function, degradation and recycling of proteins in the native cellular environment. This is also true for the field of autophagy, where tagging of microtubule-associated protein 1 light chain 3 (LC3), a protein that localises to autophagosomes, with the green fluorescent protein (GFP) allowed for direct visualisation of GFP-LC3 (and a range of other proteins, e.g. YFP-Atg5 and GFP-Atg18) in living cells [Klionsky et al., 2008]. LC3 is a ubiquitin-like protein that is essential for autophagosome formation. It is synthesised as a precursor (proLC3) and is proteolytically processed during autophagy, producing LC3-I, part of which is then modified by phosphatidylethanolamine, producing LC3-II, the form of the protein that binds autophagic membranes [Kabeya et al., 2000]. LC3-II is found on pre-autophagic membranes and autophagosomes, a property that makes it an autophagosome marker for immunochemical detection applications [Klionsky et al., 2008]. A fusion construct of LC3 with GFP, GFP-LC3, has been successfully used to directly observe autophagosome formation, autophagic degradation of substrate proteins, in vitro autophagic activity in wild-type and mutant fibroblasts as well as in vivo in whole organisms.

Despite its usefulness, several groups have identified problems with using GFP-LC3 as an autophagic membrane marker. Kuma et al. 2007 reported that GFP-LC3 can be incorporated into protein aggregates non-specifically, for example co-expression of GFP-LC3 with poly-glutamine (poly-Q) proteins. In the same study large GFP-LC3 aggregates could be observed in senescent Atg5-negative cells [Kuma et al., 2007]. In addition, permeabilisation of cells with detergents can lead to punctate staining of cells by GFP-LC3 [Ciechomska and Tolkovsky, 2007]. These artefacts of LC3 staining can to some extent be dealt with by using tandem RFP-GFP tagged LC3 [Kimura et al., 2007], careful use of controls [Tanida et al., 2008], by using proteins other than LC3 as autophagic markers [Mizushima et al., 2003], and by using indirect immunofluorescent detection of LC3 [for detailed review, see Atwal et al., 2007].

In a previous work on neurotransmitter transporter assembly, we have shown that GABA transporter molecules interact with calnexin, a chaperone resident in the endoplasmic reticulum (ER), and are targeted to organised smooth endoplasmic reticulum (OSER) membrane inclusion bodies inside the cells [Korkhov et al., 2008]. Overexpression of calnexin strongly induced proliferation of OSER membrane stacks (this has been observed with a range of ER-resident membrane proteins [Snapp et al., 2003]). However, the same compartments were also found in non-transfected cells and could be identified by antibody staining of endogenous calnexin [Korkhov et al., 2008]. A diverse range of polytopic membrane proteins was subject to targeting into these multilamellar compartments. A seemingly reasonable conjecture resulting from that work was that OSER membranes could serve as a depot for misassembled overproduced proteins, awaiting en bloc degradation, for example by autophagy. This hypothesis was attractive in the light of the reported appearance of similar multilamellar bodies in autophagy-deficient Atg7-negative cells [Komatsu et al., 2005]. I have therefore investigated whether the multilamellar bodies formed by OSER membranes are related to the autophagic pathway of protein degradation.

Here, I show that, despite their labelling by the autophagy marker GFP-LC3, OSER membranes are not subject to autophagic or lysosomal degradation pathway and are not directly related to autophagy. Targeting of GFP-LC3 to OSER is an artefact that has to be considered when analysing GFP-LC3 localisation.

## MATERIALS AND METHODS

### Reagents, DNA Constructs and Cell Lines

Earle's Balanced Salt Solution (EBSS) was purchased from Sigma. DMEM and standard cell-culture reagents were from GIBCO. 3-Methyladenine and chloroquine were from Sigma; MG132 and cycloheximide were from Calbiochem. YFP-Atg5 was provided by Dr. F. Takeshita (Yokohama, Japan); GFP-LC3 was provided by Dr. T. Yoshimori (Osaka, Japan); CFP-LC3, GFP-LC3ΔG, HcRed-LC3 and HcRed-LC3ΔG were provided by Dr. I. Tanida (Tokyo, Japan); GFP-Htt73 was provided by Dr. A. Bertolotti (Cambridge, UK). Atg5−/− MEFs were provided by Dr. N. Mizushima (Tokyo, Japan).

### Cell Culture

HEK293 cells and MEFs were cultured under normal mammalian cell culture conditions, at 37°C, supplemented with 5% and 10% CO_2_, respectively. Starvation of cells was performed by exchanging complete DMEM with EBSS medium for 24 h. Transfections were performed using Lipofectamine-2000 (Invitrogen), according to supplier's instructions, with the only modification for starved cells: EBSS was used instead of serum- and antibiotic-free DMEM medium and EBSS was used as plating medium (2 ml per well in a 6-well plate). A total of 2–4 µg of plasmid DNA was used per transfection.

### SDS–PAGE and In-Gel Fluorescence

HEK293 cells transfected with the constructs of interest, GFP-LC3 and calnexin-mCherry, were grown in 6-well or 24-well plates. Following the treatment with various chemical agents, as indicated in the figures, figure legends and text, the cells were harvested, washed in phosphate-buffered saline (PBS), resuspended in ice-cold PBS supplemented with Complete protease inhibitor cocktail (Roche) and sonicated by two pulses at 50% output using a micro ultrasonic cell disruptor (Kontes). After the SDS–PAGE of the lysates on a 4–20% gradient Tris–glycine gel (Invitrogen; equal amount of lysate protein, 25 µg/lane, as determined by amido-black assay, were loaded on the gel), the gel was removed from the cassette and scanned using variable mode imager, Typhoon 8610 (Amersham Biosciences). The settings for GFP imaging were: excitation green (532), PMT 600, emission filter 526 SP, normal sensitivity; for mCherry imaging: excitation red (633), PMT 600, emission filter 670 BP30, normal sensitivity (similar results were obtained using green laser excitation with emission filter 610 BP30). The images were analysed using ImageQuant v.3 (Amersham Biosciences).

### Confocal Microscopy

Confocal microscopy was done as described previously [Korkhov et al., 2008]. Briefly, cells were grown on poly-l-glutamine-coated (Sigma) glass coverslips until 50–80% confluence, followed by transfection and/or fixation. Cells were fixed with 4% para-formaldehyde (Sigma) in PBS, permeabilised for 30 min at room temperature using PBS with 1% BSA and 0.01% Triton X-100 and stained for 1 h with primary and secondary antibodies (in PBS, 1% BSA) where appropriate. Coverslips with stained cells were washed four times in PBS and mounted onto glass slides in Vectashield medium (Vector Laboratories) for microscopy. Images were acquired using Zeiss LSM 510 confocal microscope with a 63× objective lens. Calnexin was detected using either a mouse monoclonal FITC-conjugated anti-calnexin antibody (BD Biosciences), or a rabbit polyclonal anti-calnexin antibody (Stressgen) followed by a Texas Red-conjugated secondary anti-rabbit antibody (Invitrogen). LC3 was detected using a primary rabbit polyclonal LC3 antibody (Novus Biologicals) and a secondary anti-rabbit antibody as above. Excitation (ex.) and emission (em.) filter settings were: CFP—ex. 405 nm, em. LP 420; GFP and FITC—ex. 488 nm, em. LP 505; YFP—ex. 514 nm, em. BP 560–615; Texas Red, HcRed and mCherry—ex. 543 nm, em. BP 560–615.

## RESULTS

### GFP-LC3 Colocalises With Calnexin in the OSER Membranes in Mammalian Cells

**Fig. 1:**
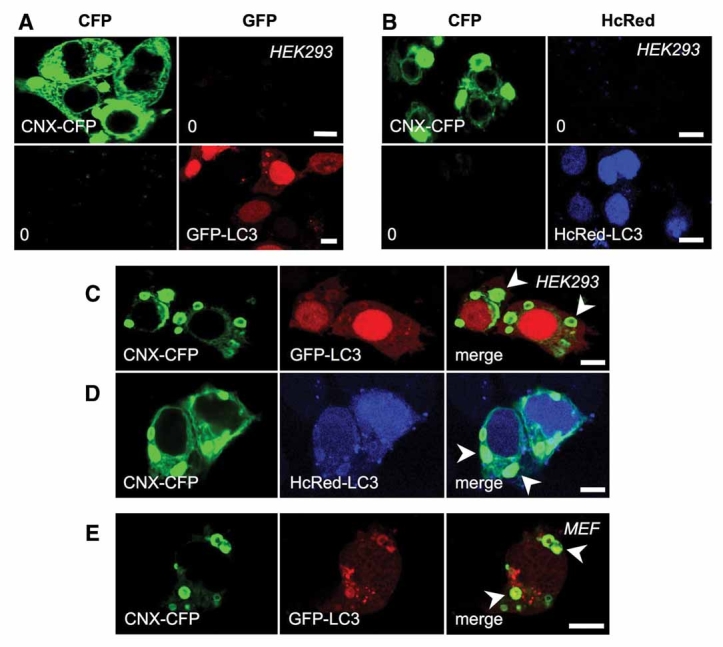
GFP-LC3 is targeted into calnexin-positive OSER membranes. A: HEK293 cells were transfected with either calnexin-CFP (CNX-CFP) or GFP-LC3 construct and subjected to confocal microscopy, as described under ‘Materials and Methods’ Section (CNX-CFP, n = 36; GFP-LC3, n = 59). B: Same is in (A), but HcRed-LC3 was expressed instead of GFP-LC3; the excitation/emission conditions were changed accordingly (CNX-CFP, n = 41; HcRed-LC3, n = 19). C,D: Co-expression of calnexin-CFP (CNX-CFP) with GFP-LC3 (n = 112) and HcRed-LC3 (n = 24), respectively, in HEK293 cells, followed by fluorescence microscopy, as described under ‘Materials and Methods’ Section reveals colocalisation of the two proteins in the multilamellar bodies. E: Same as (C), but mouse embryo fibroblasts (MEF) were transfected instead of HEK293 cells (n = 25). Scale bar is 10 µm in each image. Arrowheads indicate colocalisation of indicated proteins.

The stacks of OSER membranes are highly organised sub-organellar compartments, yet their function remains elusive. A possible specialisation for such structures could be storage and bulk disposal/utilisation of unwanted proteins and/or organelles via an autophagy-like pathway. When expressed alone, GFP-LC3 occasionally labelled large ring-shaped membrane structures (data not shown); number of cells in which such structures were observed was 24 and 17 cells for GFP-LC3-transfected HEK293 cells and mouse embryo fibroblasts (MEFs), respectively. These structures were morphologically similar to the calnexin-positive OSER membranes [Korkhov et al., 2008]. To test whether calnexin-induced OSER inclusions are pre-autophagic or autophagic, calnexin-CFP and fluorescent protein-tagged LC3 (GFP-LC3 and HcRed-LC3 [Tanida et al., 2008]) were co-expressed in HEK293 cells and in primary cultures of MEFs (Fig. 1). The cells were observed using confocal fluorescence imaging under conditions of minimal bleed-through between the CFP, GFP and HcRed channels (Fig. 1A,B).

As shown in Figure 1C,E, in cells co-expressing calnexin and GFP-LC3, the two constructs were colocalised in the concentric membrane bodies, regardless of the cell type employed. The same result was obtained when calnexin-CFP and HcRed-LC3 were co-expressed—the two protein fusions colocalised in the multilamellar membranous structures (Fig. 1D). CFP and HcRed fluorescent proteins are very well spectrally separated, displaying little or no spectral overlap (consistent with the control experiment in Fig. 1B); this precludes detection of the CFP signal through the red filters used for HcRed detection. The intensity of the GFP-LC3 or HcRed-LC3 within the OSER membranes varied from cell to cell (possibly reflecting the total cellular expression levels), but was higher than cytosolic GFP-LC3, suggestive of specific targeting of the LC3 fusion proteins into the calnexin-induced OSER.

### Endogenous LC3 Is Excluded From OSER

**Fig. 2:**
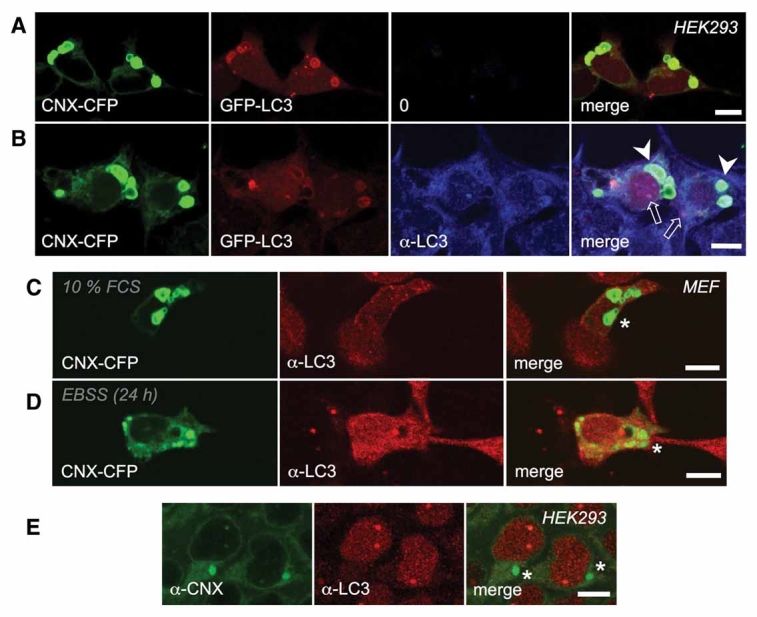
Endogenous LC3 is excluded from OSER. A: HEK293 cells were co-transfected with calnexin-CFP and GFP-LC3 and imaged as described in Figure 1, with an additional image acquired through a red channel, to show minimal bleed-through from the GFP-channel into the red one (n = 19). B: Same as in (A), but after labelling of LC3 with an anti-LC3 antibody (α-LC3; detected by Texas Red anti-rabbit), as described under ‘Materials and Methods’ Section (n = 53). C: Mouse embryo fibroblasts transfected with calnexin-CFP (CNX-CFP) and stained with an anti-LC3 antibody, as in (A), show exclusion of endogenous LC3 (n = 24); here and in all following images, asterisks indicate lack of colocalisation of indicated proteins/antibodies, unless specified otherwise. D: Same as in (C), but after autophagy induction by incubation in EBSS for indicated time period (n = 21). E: Co-staining of HEK293 cells with an anti-calnexin (α-CNX) and anti-LC3 (α-LC3) antibody reveals lack of colocalisation of endogenous calnexin and endogenous LC3 within the multilamellar bodies (n = 21; the number cells with observed OSER structures represented 10% of the total observed cell population); upon 6 h incubation in EBSS medium, 11 out of 97 cells were found positive for multilamellar membrane inclusions labelled an antibody for the endogenous calnexin (not shown). Scale bar is 10 µm in each image. Arrowheads indicate colocalisation of indicated proteins in OSER; open arrows indicate colocalisation in punctate structures. Asterisks indicate lack of colocalisation of indicated fluorescent protein fusions/antibody-stained proteins.

The data described in Figure 1 were suggestive of a possible link between autophagy and OSER membranes. However, given the possible artefacts arising from GFP-LC3 imaging for autophagic membrane detection [Klionsky et al., 2008], I tested whether endogenous LC3 would also be targeted to calnexin-positive multilamellar bodies. The control experiments revealed that the anti-LC3 antibodies are able to detect the ectopically expressed GFP-LC3 protein within the multilamellar bodies (Fig. 2A,B; absence of cross-talk between GFP and red fluorescence channels is shown in Fig. 2A). Incubation of cells in nutrient-deprived media is known to potently induce autophagic protein degradation [Lee et al., 1989; Klionsky et al., 2008]. During autophagy LC3 is proteolytically processed, producing LC3-I, part of which is then modified by phosphatidylethanolamine, producing LC3-II, the form of the protein that binds autophagic membranes [Kabeya et al., 2000]. To test whether a processed form of GFP-LC3, GFP-LC3-II, was attracted to the OSER, cells were imaged after growth under standard culture conditions (DMEM supplemented with foetal calf serum) or after starvation by incubation in Earle's Balance Salt Solution (EBSS) to induce autophagy. MEFs (as well as HEK293 cells) showed complete exclusion of endogenous LC3 from calnexin-CFP-induced OSER (Fig. 2C). Similarly to cells under normal culture conditions, starved cells showed exclusion of LC3 from calnexin-induced multilamellar bodies (Fig. 2D). Furthermore, co-staining of non-transfected HEK293 cells showed exclusion of LC3 from OSER stacks (Fig. 2E), which was independent of the autophagy induction (not shown).

### Atg5 Is Excluded From OSER

**Fig. 3:**
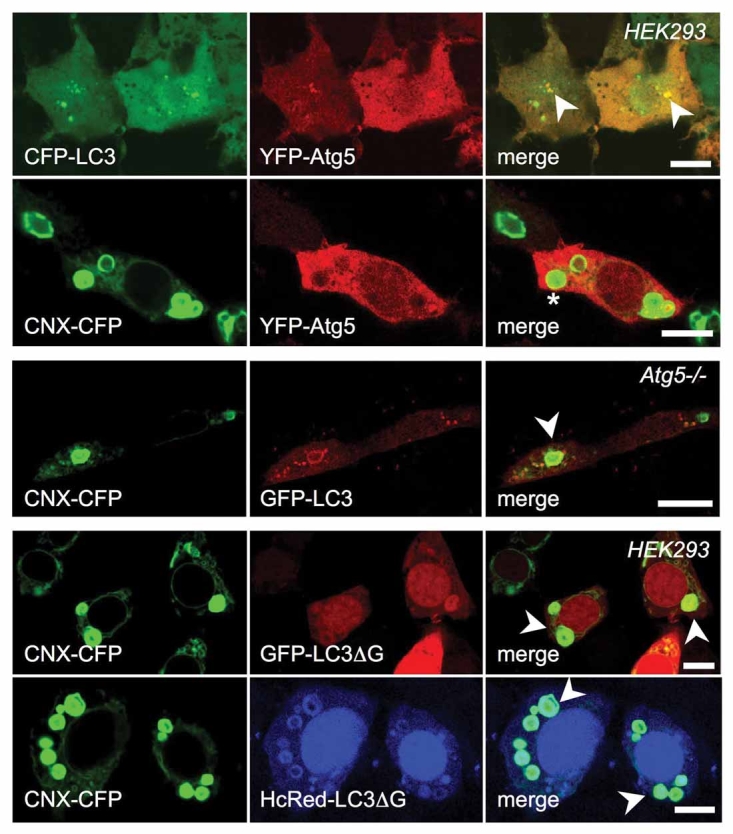
Calnexin-induced OSER membranes are labelled by GFP-LC3 independently of autophagy. A: YFP-Atg5 colocalises with the co-expressed CFP-LC3 in punctate structures in HEK293 cells (n = 11); the same result was observed after 24 h incubation in EBSS medium (not shown, n = 13). Similar results were obtained by imaging YFP-Atg5 and HcRed-LC3 (not shown, n = 26). B: YFP-Atg5 is excluded from multilamellar bodies induced by calnexin-CFP overexpression in HEK293 cells (n = 37; asterisk indicates lack of colocalisation of indicated fluorescent protein). C: Mouse embryo fibroblasts negative for Atg5 (Atg5−/−) were co-transfected with calnexin-CFP and GFP-LC3 (n = 26). D,E: Processing-defective mutant, GFP-LC3ΔG (n = 39) and HcRed-LC3ΔG (n = 26) is targeted to the OSER membranes. Scale bar is 10 µm in each image. Arrowheads indicate colocalisation of indicated proteins in OSER.

Because of the conflicting results of experiments with GFP-LC3 and antibody-stained endogenous LC3, I tested the colocalisation of calnexin with Atg5, another protein known to occur at the autophagic membrane [Mizushima et al., 2001]. YFP-Atg5 expressed alone displayed predominantly diffuse cytosolic distribution pattern, forming puncta in a fraction of the cells; these punctate structures were positive for CFP-LC3 [Tanida et al., 2008], indicating that the YFP-Atg5 targeting to the autophagic membranes is preserved (Fig. 3A). When YFP-Atg5 was co-transfected together with calnexin-CFP in HEK293 cells, the calnexin-positive multilamellar membranes did not stain for Atg5 (Fig. 3D). This result paralleled the data obtained with endogenous LC3 as well as the previous results of co-expression of calnexin with cytosolic proteins or fluorescent proteins alone [Korkhov et al., 2008]. Starvation of cells did not lead to any detectable colocalisation of calnexin with Atg5 in the multilamellar bodies (data not shown). This observation ruled out the possibility that autophagy induction was required for targeting of YFP-Atg5 into the OSER membranes.

### GFP-LC3 Colocalises With Calnexin in Multilamellar Structures in Atg5-Negative Cells

Results with YFP-Atg5 as an alternative autophagosome marker lent support to the antibody-staining experiments, further indicating that OSER membranes have no direct connection to autophagy. I then investigated whether the calnexin/GFP-LC3 colocalisation within multilamellar bodies would be preserved in Atg5-negative background. Co-expression of GFP-LC3 with calnexin in the Atg5−/− MEFs revealed that the two proteins colocalised in multilamellar bodies in these cells (Fig. 3C; colocalising structures are indicated by arrowheads). This finding provided a strong evidence for the interpretation that GFP-LC3 was spuriously targeted to the OSER.

### Fluorescent Protein-LC3ΔG Fusion Localises to the OSER Membranes

Targeting of LC3 (and GFP-LC3) to the autophagic membranes is dependent on its correct processing—proteolysis and lipid modification. For this process, a glycine-120 residue of LC3 is required; LC3ΔG constructs, in which glycine-120 have been deleted, fail to be processed and targeted to the autophagic vesicles [Tanida et al., 2004, 2008]. I co-expressed calnexin-CFP with GFP-LC3ΔG and HcRed-LC3ΔG and performed fluorescence imaging under identical conditions to those used in Figure 1. As shown in Figure 3D,E, the OSER membranes were positive for GFP-LC3ΔG and HcRed-LC3ΔG. This provided a strong indication that the fluorescent protein-LC3 fusion targeting to the OSER is independent of LC3 lipidation (which is a prerequisite for LC3 recruitment to the autophagic membranes).

### Aggregates of Huntingtin With a 73-Glutamine Expansion Are Excluded From OSER Membranes

**Fig. 4:**
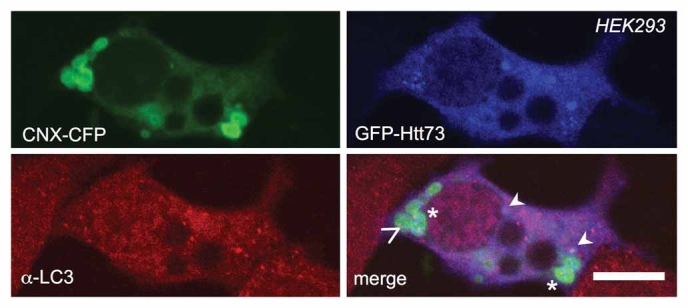
Poly-Q expanded huntingtin fragment is excluded from OSER membranes. HEK293 cells growing on coverslips were co-transfected with calnexin-CFP (CNX-CFP) was and a 73-glutamine-expanded fragment of huntingtin tagged with GFP (as described in ‘Results’ Section). After 24 h in culture cells were fixed, permeabilised and stained with an anti-LC3 antibody for immunofluorescence imaging (n = 10). No colocalisation of either GFP-Htt73 or endogenous LC3 was observed (indicated by asterisks). In some instances, small punctate structures positive for all three proteins were found (indicated by arrowheads); in others, puncta with only GFP-Htt73 or only LC3 could be observed (V-shaped arrowhead), often surrounded by the multilamellar bodies. Scale bar is 10 µm.

Taken together, the data indicated that targeting of GFP-LC3 targeting into OSER membranes was unrelated to the role of LC3 in autophagy. Accordingly, I selected a model protein unrelated to biogenesis of autophagic compartments (but otherwise known to be targeted into autophagosomes), namely, a poly-Q-expanded version of huntingtin, the protein involved in Huntington's disease. This protein has been observed in the autophagic vesicles [Atwal et al., 2007] and is known to be subject to autophagic degradation [Qin et al., 2003]; modulation of autophagy has been proposed as a possible therapeutic treatment to clear huntingtin aggregates in disease models [Ravikumar et al., 2004]. Furthermore, poly-Q aggregates have been previously shown to incorporate GFP-LC3 [Kuma et al., 2007] as well as endogenous LC3 [Kouroku et al., 2007]. To test whether the mechanism of GFP-LC3 targeting to the OSER membranes is related to that of poly-Q and GFP-LC3 or LC3 colocalisation, I co-expressed calnexin and GFP-Htt73, a fragment of 163 N-terminal huntingtin residues with a 73 poly-Q expansion [Rousseau et al., 2004]. Imaging of endogenous LC3 together with transiently co-expressed calnexin-CFP and GFP-Htt73 in HEK293 cells revealed the presence of punctate structures positive for all three proteins, or with no colocalisation between any of the three (Fig. 4, arrowheads indicate colocalisation; V-shaped arrowheads indicate lack of colocalisation in punctate inclusions). However, both GFP-Htt73 and endogenous LC3 were excluded from calnexin-induced OSER stacks in transfected HEK293 cells (Fig. 4). Electron microscopic characterisation of the OSER membranes shows that these compartments (and the protein components therein) are highly ordered, as opposed to the aggresomes of the huntingtin fragments [Firdaus et al., 2006]. The experiment with calnexin-CFP and GFP-Htt73 shows that the OSER membranes induced by calnexin overexpression are distinct from the huntingtin aggregates and thus the mechanism of GFP-LC3 recruitment into these structures is likely to be different.

### Calnexin Is Not Subject to Autophagic or Lysosomal Degradation

**Fig. 5:**
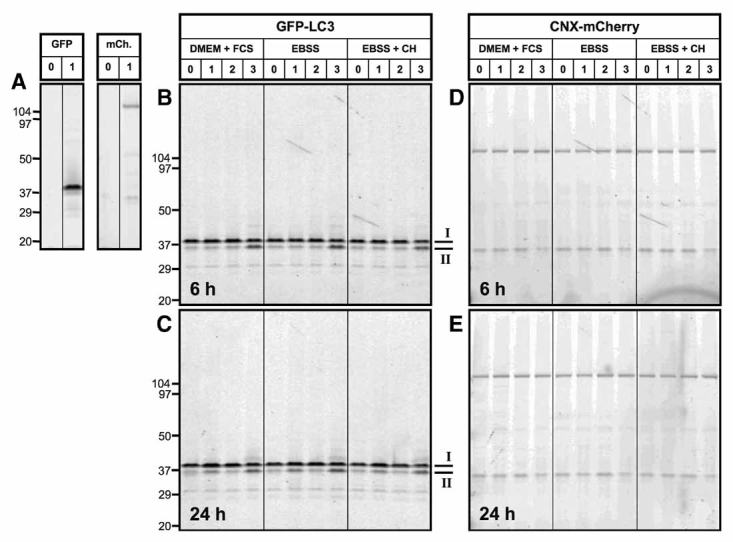
Calnexin is not subject to autophagic degradation. A: GFP-LC3- and calnexin-mCherry-transfected HEK293 cell lysates were separated on an 4–20% Tris–glycine SDS–PAGE gel, followed by in-gel GFP (‘GFP’) and mCherry (‘mCh.’) fluorescence detection, as in ‘Materials and Methods’ Section. B–E: An SDS–PAGE gel with lysates of HEK293 cells co-expressing GPF-LC3 (B,C) and calnexin-mCherry (D,E) scanned using the corresponding channels. Cells cultured in complete DMEM with 10% serum are indicate by ‘DMEM + FCS’; starved cells and cells with 35 µM cycloheximide are indicated by ‘EBSS’ and ‘EBSS + CH’, respectively. Lanes with no drug treatment is indicated by ‘0’; ‘1’, ‘2’, and ‘3’ indicate presence of 10 µM MG132, 2 mM 3-methyladenine and 100 µM chloroquine, respectively. Panels B and D show data on cells culture for 6 h in the presence of the indicated drugs, whereas C and E show data upon 24 h incubation. Equal amount of total lysate protein were loaded (25 µg/lane). GFP-LC3-I and the processed form, GFP-LC3-II, are marked as bands ‘I’ and ‘II’, respectively; the full-length calnexin-mCherry band is labelled by an asterisk. The experiment was performed twice.

A predominant portion of overexpressed calnexin-fluorescent fusion protein is found in the OSER. Therefore, I tested whether calnexin molecules, and thus the OSER compartments, are subject to proteolysis by either ER-associated degradation (ERAD), or autophagic/lysosomal degradation pathway. For this purpose, HEK293 cells were transfected with GFP-LC3 and calnexin-mCherry constructs. The lysates of the cells expressing either one or both of these proteins were subjected to SDS–PAGE followed by in-gel fluorescence, as described under ‘Materials and Methods’ Section. Lysates of non-transfected cells showed minimal background fluorescence in both GFP and mCherry (Fig. 5A; lane 0—non-transfected cells; lane 1—cell transfected with an indicated construct).

The same methodology was applied to lysates of HEK293 cells co-expressing GFP-LC3 and calnexin-mCherry cultured in presence of chemicals modulating various protein degradation pathways. As shown in Figure 5B,C, processing of GFP-LC3-II occurred even in non-starved cells at steady state, judged by the presence of a cleavage product under the 41 kDa band of the full-length GFP-LC3 (Fig. 5B,C; lane 0 in each panel). Incubation with an autophagy inhibitor, 3-methyladenine lead to modest accumulation of the unprocessed GFP-LC3 (Fig. 5B,C; lane 2). The processed product was not significantly affected by incubation of cells with autophagy inhibitor, 3-methyladenine (Fig. 5B,C; lane 2 in each panel), but was enhanced after 24 h incubation with MG132, a potent proteasome inhibitor (Fig. 5B; lane 1). An even stronger enhancement of cleaved GFP-LC3 accumulation, in addition to appearance of higher molecular weight conjugates of GFP-LC3, was evident already after 6 h incubation with chloroquine, and inhibitor of lysosomal degradation (Fig. 5B,C). Similar results were obtained with starved cells in the presence and in the absence of translation inhibitor, cycloheximide (Fig. 5B,C; panels ‘EBSS’ and ‘EBSS + CH’, respectively).

The expression levels of the full-length calnexin-mCherry construct co-expressed with the GFP-LC3, remained unchanged under all experimental conditions (Fig. 5D,E). The only significant difference was observed upon long-term ERAD inhibition by MG132 (Fig. 5E; lane 1), where accumulation of partially digested fragments with molecular weight in the range of 90–100 kDa could be detected. No significant change in the amount of calnexin-mCherry was detected upon induction of autophagy and treatment of cells with 3-methyladenine or chloroquine (Fig. 5D,E; lanes 2 and 3). Furthermore, even prolonged inhibition of total protein synthesis in presence of cycloheximide caused no dramatic changes in calnexin levels (Fig. 5E; lane 3). These results altogether suggest that the calnexin-positive OSER membranes are not subject to bulk degradation by lysosomes.

### GFP-LC3 Targeting Into the OSER Membranes Is Not Affected by Inhibitors of Autophagic and Lysosomal Protein Degradation

**Fig. 6:**
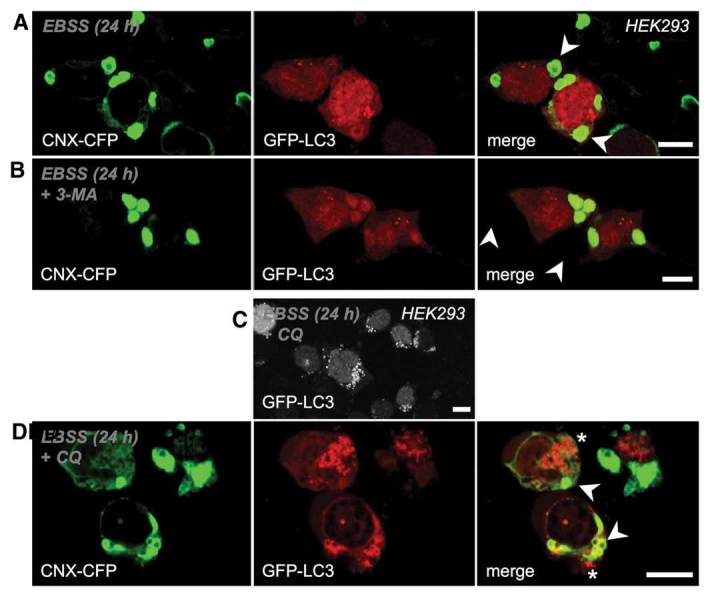
GFP-LC3 localisation to OSER is insensitive to autophagy inhibitors. A: HEK293 cells co-expressing calnexin-CFP and GFP-LC3 were cultured for 24 h in EBSS medium and imaged as described for Figure 1 (n = 22); the same result was observed after 6 h incubation (not shown, n = 41). B: Upon incubation in EBSS medium for 24 h in presence of 3-methyladenine (‘3-MA’), the localisation of GFP-LC3 to OSER membranes was preserved (n = 30); the same result was observed after 6 h incubation (not shown, n = 38). C: Cells expressing GFP-LC3 were starved and treated with 100 µM chloroquine (‘CQ’) to inhibit the lysosomal degradation (n = 36). D: Same as (C), with cells co-expressing calnexin-CFP and GFP-LC3 (n = 29). Scale bar is 10 µm in each image. Arrowheads indicate colocalisation of calnexin-CFP and GFP-LC3 in the OSER membranes; asterisks indicate lack of colocalisation of these proteins in the accumulated lysosomes.

To obtain a morphological correlate of the in-gel fluorescence data, I performed imaging of the cells expressing calnexin-CFP and GFP-LC3 after starvation, in presence of 3-methyladenine or chloroquine. As expected, no the colocalisation of calnexin-CFP and GFP-LC3 was preserved in cells after 6 h (not shown) or 24 h incubation in EBSS medium in the absence (Fig. 6A) or in the presence of 3-methyladenine (Fig. 6B).

Incubation of GFP-LC3-expressing HEK293 cells lead to accumulation of GFP-LC3-positive lysosomes in all transfected cells (Fig. 6C). Co-expression of calnexin-CFP and GFP-LC3 did not prevent colocalisation of the two in the OSER structures. However, the punctate lysosomes positive for GFP-LC3 were negative for calnexin (Fig. 6D). This result confirmed the data on calnexin insensitivity to lysosomal degradation inhibition, lending further support to the notion that OSER membranes are not substrates for autophagy.

## DISCUSSION

ER-derived multilamellar bodies, or OSER, have been observed in cell culture, in untransfected cells and upon protein overexpression [Snapp et al., 2003; Korkhov et al., 2008]. Similar inclusions have also been observed in mice upon deletion of genes essential for autophagy [Komatsu et al., 2005]. A recent report also described accumulation of such structures in neurons upon traumatic brain damage [Lai et al., 2008]. Furthermore, multilamellar bodies are observed in people with some genetic diseases (e.g. torsion dystonia [Gonzalez-Alegre and Paulson, 2004] and Emery-Dreifuss disease [Fidzianska et al., 2004]). The physiological and pathological significance of the OSER membrane stacks is unclear. These multilamellar membrane structures could either be toxic to cells, or they could mitigate the toxicity of the erroneously assembled proteins that they sequester. These membrane structures are continuous with the ER, based on FRAP experiments in living cells [Okiyoneda et al., 2004], a property that sets them apart from inclusion bodies, such as the ones containing mutant α_1_-anti-trypsin [Granell et al., 2008]. Recently, two separate ER-associated protein degradation pathways have been proposed: (a) classical ubiquitin/proteasome system-mediated degradation (ERAD-I), and (b) autophagy/lysosomal pathway (ERAD-II) [Fujita et al., 2007]. Because a broad range of proteins can induce OSER stacks, it has been tempting to hypothesise that the function of such compartments should be related to storage and en masse disposal of overproduced ‘junk’ proteins by an ERAD-II-like mechanism. However, the present results argue strongly against this possibility. Perhaps a more likely alternative for the multilamellar OSER stacks is to remain quiescent, abstaining from any downstream degradation. In line with this hypothesis, Bernales et al. 2006 have recently shown that yeast cells cope with ER membrane proliferation during unfolded protein response (UPR) by sequestering them into large inclusions, similar to OSER lamellar bodies, even in the absence of vacuolar proteolysis. A similar model applied to the OSER membranes would be consistent with the results of the present work, including imaging of individual cells and measurements of protein levels in the whole population of the cells. Thus, an evolutionary connection may exist between the OSER membranes in mammalian cells and the adaptation to UPR in yeast; both could represent a common conserved mechanism that eukaryotic cells use to deal with excessive or erroneous protein biosynthesis.

The observation of GFP-LC3 labelling of the OSER multilamellar bodies is in line with previously published reports of artefactual incorporation of LC3 into intracellular structures other than autophagosomes [Ciechomska and Tolkovsky, 2007; Kuma et al., 2007]. Here, I show that ER-derived multilamellar bodies represent yet another compartment without a direct connection to autophagy that can be visualised by GFP-LC3. However, an important distinction from the findings of Kuma et al. 2007 concerning incorporation of GFP-LC3 by poly-Q aggregates is that, while protein density within OSER membranes is high, the proteins therein are not aggregated and are fully mobile in the membrane plane [Korkhov et al., 2008] (e.g. an aggregation-prone CFTR mutant, ΔF508, is in a fully mobile, non-aggregated state within multilamellar bodies; unpublished observation). Thus the targeting of GFP-LC3 to the multilamellar membranes may not be ascribed to non-specific protein aggregation. It is possible that the calnexin-enriched ER membranes share some similarity with the autophagic membranes, allowing for GFP-LC3 recruitment. However, taking into account the experiments described in Figures 2, 3, and 6, namely the lack of endogenous LC3 targeting to OSER, the recruitment of GFP-LC3ΔG into the OSER, and independence of this targeting of autophagy-modulating agents, clearly indicate that the mechanism underlying this process is different from that of LC3-II binding to autophagic membranes [Kabeya et al., 2000].

Thus, in addition to demonstrating that OSER membranes are not subject to autophagic degradation, this work points to another potential source of artefacts associated with GFP-LC3-based visualisation of autophagic membranes. Experiments involving this marker must be interpreted with caution: multilamellar bodies derived from ER represent a yet another compartment that can be labelled by GFP-LC3 without any direct relation to autophagy.
